# Numerical framework for simulating bio-species transport in microfluidic channels with application to antibody biosensors

**DOI:** 10.1016/j.mex.2020.101132

**Published:** 2020-11-06

**Authors:** Fatemeh Shahbazi, Masoud Jabbari, Mohammad Nasr Esfahani, Amir Keshmiri

**Affiliations:** a Department of Mechanical, Aerospace and Civil Engineering, University of Manchester, Manchester M13 9PL, UK; b Department of Electronic Engineering, University of York, York YO10 5DD, UK; c Manchester University NHS Foundation Trust, Manchester Academic Health Science Centre, Southmoor Road, Wythenshawe, Manchester M13 9PL, UK

**Keywords:** Microfluidics, Biosensors, CFD, Computational fluid dynamics, Computational code, Numerical modelling

## Abstract

Diagnosis is a fundamental stage in health care and medical treatment. Microfluidic biosensors and lab-on-a-chip devices are amongst the few practical tools for achieving this goal. A new computational code, specifically for designing microfluidic-integrated biosensors is developed, the details of which is presented in this work. This new approach is developed using control-volume based finite-element (CVFEM) method and solves bio-recognition chemical reactions and full Navier–Stokes equations. The results of the proposed platform are validated against the experimental data for a microfluidic based biosensor, where excellent agreement is achieved. The properties of the biosensor, sample, buffer fluid and even the microfluidic channel can easily be modified in this platform. This feature provides the scientific community with the ability to design a specific biosensor for requested point-of-care applications.

• A new approach is developed using control-volume based finite-element (CVFEM) method for investigating flow inside a microfluidic-integrated biosensor. It is also used to study the influence of surface functionalization on binding cycle.
• The proposed model solves bio-recognition chemical reactions as well as full Navier–Stokes and energy equations. Experimental-based or personalized equations of the chemical reactions and flow behaviour are adoptable to this code.
• The developed model is Fortran-based and has the potential to be used in both industry and academia for biosensing technology.

## Graphical abstract

**Figure:**
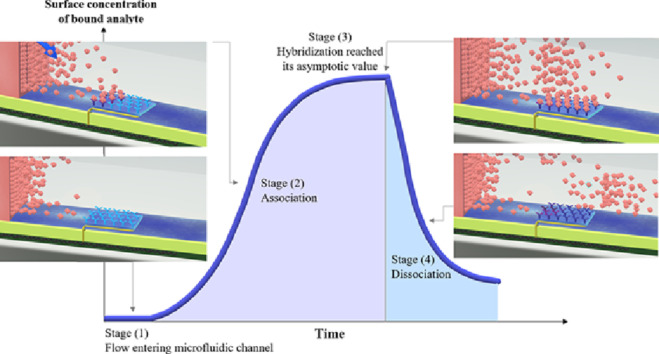

**Table:** Specifications Table

Subject Area:	Engineering
More specific subject area:	*Biosensing, Biomedical Engineering, Mechanical Engineering*
Method name:	*Convective-diffusive-Langmuir transport simulation of bio-species inside a microfluidic-integrated biosensor with an implicit control-volume based finite-element method (CVFEM)*
Name and reference of original method:	*Langmuir reaction; control-volume based finite-element method (CVFEM); Navier-Stokes equation; Fick's law; and Karimian-Schneider method*
Resource availability:	*This method has been developed in Fortran*

## Method details

Requirements

• *Fortran language platform*
• *Operating system: Windows XP and higher/Linux/Mac*
• *High performance computing (HPC) recommended*

Reuse potential

• *This code can be used by any numerical modeller with interest in CFD*

## Introduction

**Fig. 1:**
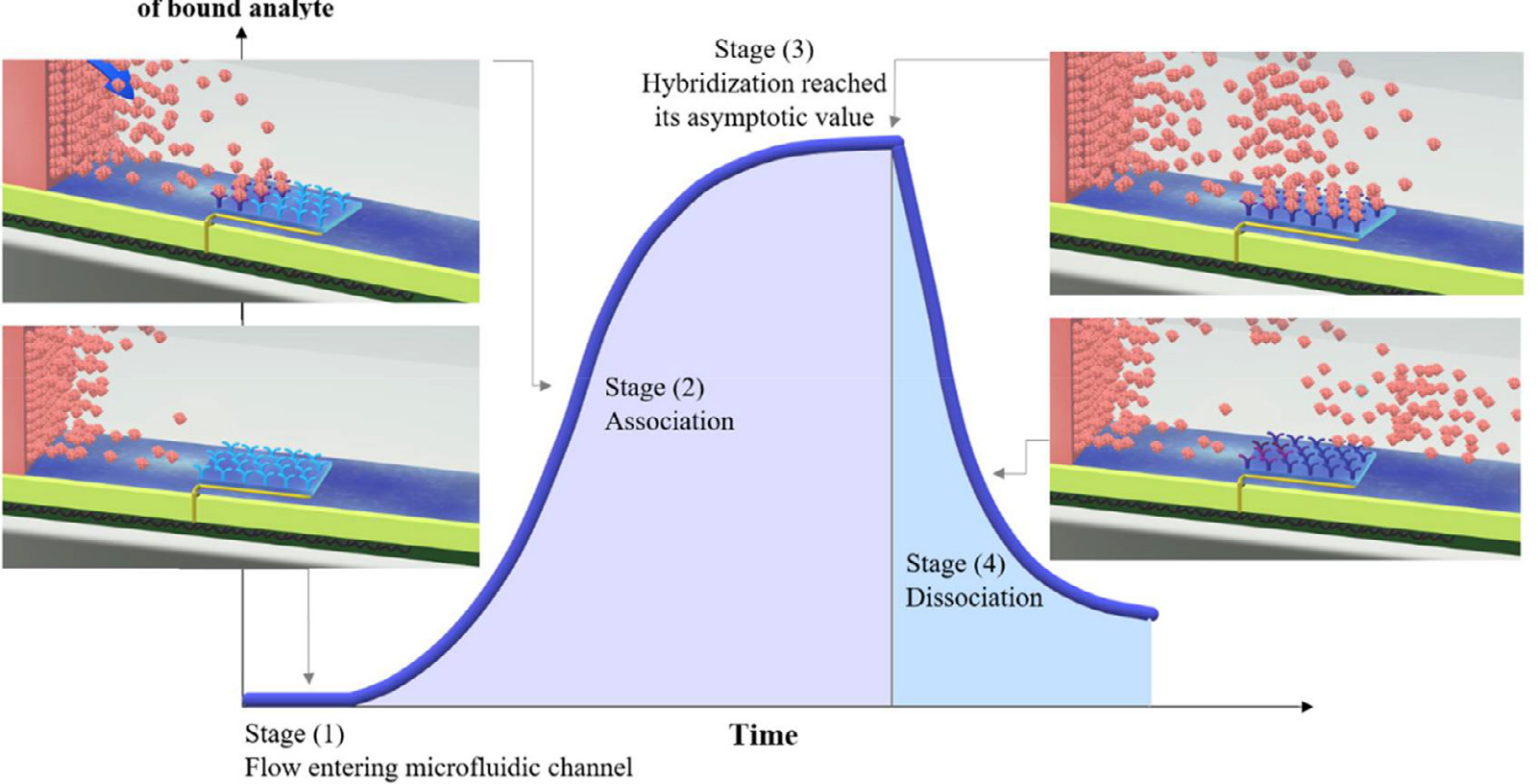
Schematic view of the four stages of the binding cycle in a microchannel equipped with heterogeneous biosensor.

Biosensors are affordable real-time monitoring devices that make dynamic monitoring of specific targeted molecules (environmental, agricultural or body micro samples) possible. According to the world health organization, in the past two decades, ischemic heart diseases, stroke, chronic obstructive, pulmonary disease, Alzheimer, lower respiratory infections are top five global causes of death [21], while they can be cured if they are detected at early stages. These examples and a lot of deadly contagious diseases, cancers and environmental causes can be detected and analysed by biosensors which would make them one of the most interesting devices in the 21st century [8]. Microfluidic-integrated biosensors are functionalized surfaces installed in a channel with the scale of micron. A schematic view of the detection process in these biosensors is illustrated in Fig. 1. Targeted molecules, which can be coronavirus or any kind of virus, DNA or protein that is needed to be detected or measured, is first mixed with the buffer fluid, which helps with a better detection of the targeted molecules. Then the buffer fluid flows into the microfluidic channel, and as it passes the functionalized surface, chemical reactions take place at this stage. The transducer converts these chemical reactions into measurable signals, which show the presence of the targeted molecule in the sample and its amount. The binding cycle of this detection process is presented in Fig. 1, which shows that in the first stage, a small portion of biological samples is inserted into the microfluidic channel (e.g. body sweat which contains ions, metabolites and hormones). In the next stage the samples react with the antibodies while passing by the surface of the biosensor. As saturation takes place in the third stage, a wash out flow goes into the channel (stage 4) and completes the binding cycle. Signals of these binding cycles get processed and reveal the results of the detection procedure.

For the purpose of designing biosensors and improving their efficiencies, various experimental works and limited number of numerical analyses have been conducted [4,[16], [17]]. In recent years, computational fluid dynamics has effectively been applied to various biomedical-related projects involving design, validation and proof-of-concept [2,^6^,^7^,[11], [12], [13],18]. Previous numerical analyses are mainly based on finite element methods (FEM) or finite volume methods (FVM). In the FVM approach, diffusion coefficient is reduced by 10^−10^ in order to reduce numerical instability [3]. On the other hand, in the FEM approach, the analytical velocity profile is used instead of solving the whole Navier–Stokes equation. Both cases lead to an almost non-realistic detection time. In the previous works, different software packages and codes were utilized to model the flow inside the microfluidic channel. The discretization method is a key component in these numerical solutions. Table 1 presents three different discretization methods; finite element method (FEM), control-volume method (CVM) and control-volume based finite-element method (CVFEM). FEM has geometrical flexibility, CVM has physical intuition and CVFEM is a powerful combination of these two methods [19].

**Table 1:** Comparison of the present platform with the existing software packages and codes.

Software packages and codes	COMSOL FEMLAB Code based on Galerkin method	CFD-ACE+Ansys-CFX, Ansys-Fluent Flow3D	Current numerical model
Numerical diffusion	Needs adjustment of the diffusion constant for avoiding the numerical diffusion	Needs adjustment of the diffusion constant for avoiding the numerical diffusion	No numerical diffusion
Discretizati-on method	Finite element method (FEM)	Control volume method (CVM)	Control-volume based finite-element method (CVFEM)
Discretization of the numerical domain			

## Methodology

**Fig. 2:**
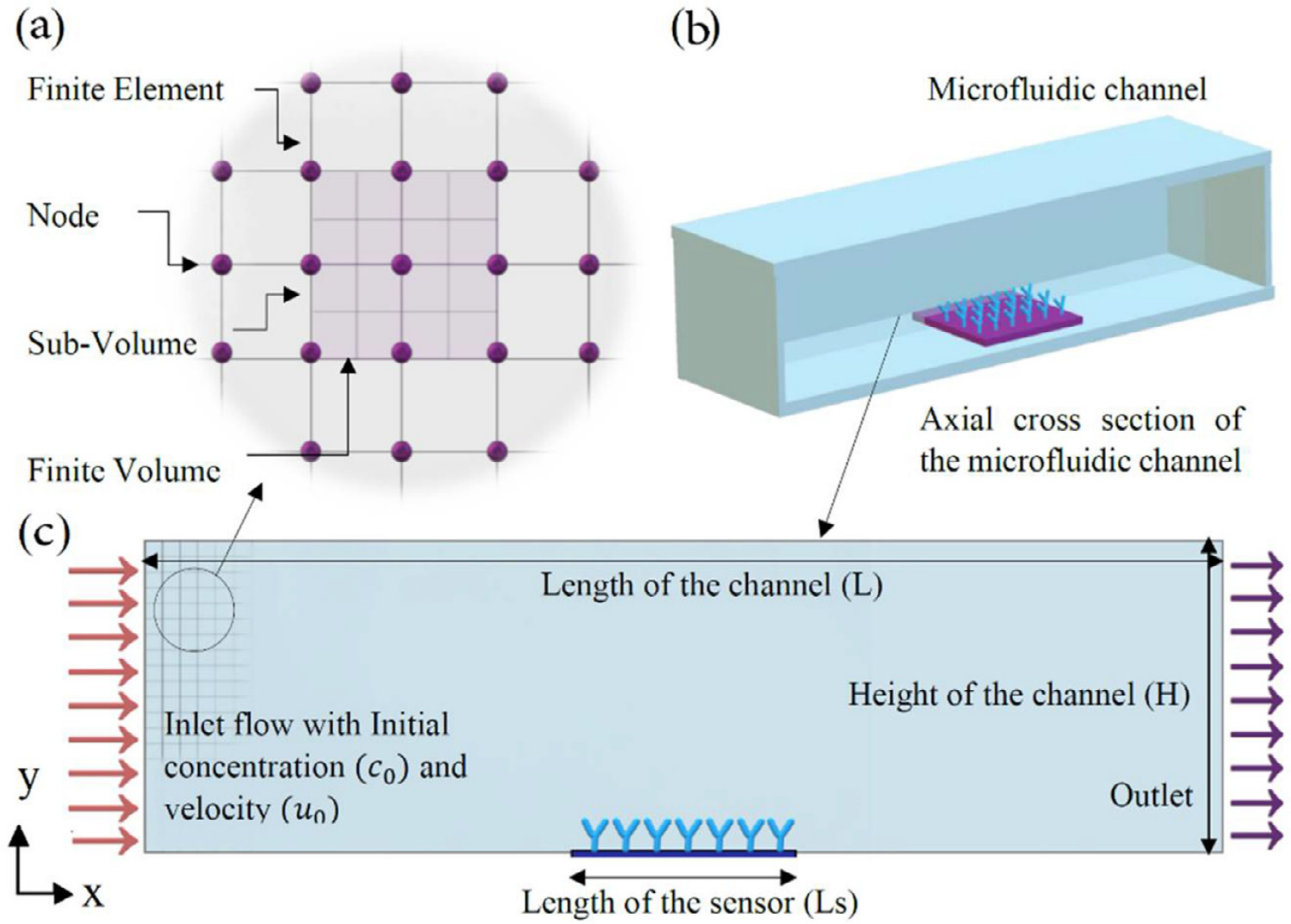
Schematic of the microfluidic-integrated biosensor along with its axial cross section, numerical domain, and boundary conditions.

In the present work, a new approach is used for conducting numerical simulations in this field which provides reliable and realistic binding cycles. Results have been validated with experimental data in the companion publication by the present authors [15]. The main challenge in numerical simulation is achieving a realistic modelling of the transport of bio-species and their reactions on the functionalized surface [10]. In order to overcome this challenge, in the current model, full Navier-Stokes equations coupled with convection, diffusion and reaction of targeted molecules Eqs. (1)–((3)) are solved implicitly with control-volume based finite-element method (CVFEM). The coupled system of equations is discretized using high order discretization methods such as the improved skewed upwind differencing (SUD) scheme. Details of the numerical domain and boundary conditions that are implemented in this model are summarized in Fig. 2 and Table 2. For concentration (*c*) of bio-species on the sensor and on the walls, ‘Homogeneous Neumann’ and ‘Neumann’ conditions are prescribed, respectively. In the present code, unsteady viscous fluid flow in a channel is solved and the flow is assumed to be laminar, incompressible and 2D. The flow is solved using the finite element-based control volume method.

**Table 2:** The Boundary conditions used in the present study.

Type	Velocity	Concentration
Interior	Navier-Stokes equations	Convection-diffusion-reaction
Walls	No slip	Homogeneous Neumann ($\frac{\partial c}{\partial n}=0$)
Sensor	No slip	Neumann ($\frac{\partial c}{\partial n}=-\frac{1}{D}\frac{\partial b}{\partial t}$)
Inlet	u=u_0_	c=c_0_
Outlet	Straight out	$\vec{n}.\left(D\nabla c\right)=0$

## Governing equations

The proposed numerical platform solves the Navier–Stokes equations Eqs. (1)–(3) to simulate the buffer fluid flow and Langmuir-Hinshelwood model (Eq. (11)) to simulate the kinetics of adsorption of molecules. The fluid is considered as continuum and incompressible [20], due to negligible changes in density.

(1) $$\frac{\partial \rho }{\partial t}+\rho \left[\frac{\partial u}{\partial x}+\frac{\partial v}{\partial x}\right]=0$$

(2) $$\rho \frac{\partial u}{\partial t}+u\frac{\partial u}{\partial x}+v\frac{\partial u}{\partial y}=-\frac{\partial p}{\partial x}+\mu \left(\frac{\partial^{2}u}{\partial x^{2}}+\frac{\partial^{2}u}{\partial y^{2}}\right)$$

(3) $$\rho \frac{\partial v}{\partial t}+u\frac{\partial v}{\partial x}+v\frac{\partial v}{\partial y}=-\frac{\partial p}{\partial y}+\mu \left(\frac{\partial^{2}v}{\partial x^{2}}+\frac{\partial^{2}v}{\partial y^{2}}\right)$$

where *u* and *v* are the velocity in the *x* and *y* direction, respectively, while *ρ* is the density and *μ* is the molecular viscosity. Continuity equation has the role of controlling pressure field in the domain, but as it is shown, there is no pressure term in continuity equation. In order to relate the pressure field effects in continuity equations, the Karimian–Schneider [5] method (Eqs. (4) to (6)) is used.

(4) $$\hat{u}=u_{BL}-\frac{1}{\beta }\left[-\rho \left(u\frac{\delta v}{\delta y}-v\frac{\delta u}{\delta y}\right)+\frac{\delta p}{\delta x}\right]$$

(5) $$\hat{v}=v_{BL}-\frac{1}{\beta }\left[-\rho \left(v\frac{\delta u}{\delta x}-u\frac{\delta v}{\delta x}\right)+\frac{\delta p}{\delta y}\right]$$

(6) $$\beta =\frac{\rho \bar{V_{j}}}{\Delta s}+\frac{\mu }{L^{2}}$$

where *BL* refers to the values in boundary layer, Δs is the area of each element, *L* is the reference length and $\bar{V_{j}}$ is the velocity of the main element. In the convection term of momentum, *u* and *v* are obtained using the physical influence scheme (PIS) method (Eqs. (7) and (8)).

(7) $$u_{j}=\frac{\rho \bar{V_{j}}}{\Delta s}\frac{1}{\beta }u_{up_{j}}+\frac{\mu }{L^{2}}\frac{1}{\beta }u_{BL_{j}}+\frac{1}{\beta }\left(-\frac{\partial P}{\partial x}\right)$$

(8) $$v_{j}=\frac{\rho \bar{V_{j}}}{\Delta s}\frac{1}{\beta }v_{up_{j}}+\frac{\mu }{L^{2}}\frac{1}{\beta }v_{BL_{j}}+\frac{1}{\beta }\left(-\frac{\partial P}{\partial y}\right)$$

For simulating the kinetics of adsorption of molecules on a functionalized solid surface (e.g. DNA hybridization and virus detection etc.), the Langmuir–Hinshelwood model [1] is used. Since targets are constantly captured by ligands and dissociate at a smaller rate, such reactions are weakly reversible. Using Fick's law (Eq. (9)) and evaluating the mass balance and effect of velocity field would give us the complete equation system for modelling the concentration (Eq. (10)). The reaction rates can be written as Eq. (11).

(9) F=−D∇c

(10) $$\frac{\partial c}{\partial t}+\vec{u}.\nabla c=\nabla .\left(D\nabla c\right)+S$$

(11) $$\frac{\partial b}{\partial t}=k_{on}c_{0}\left(b_{max}-b\right)-k_{off}b$$

where *c* is the concentration of the targeted molecules, *c*_0_ is the inlet concentration, *b* is the surface concentration of bound analyte [14], *b*_max_ is the density of binding sites on the sensor, *D* is the diffusion coefficient and *S* is the source/sink term.

## Discretization

**Fig. 3:**
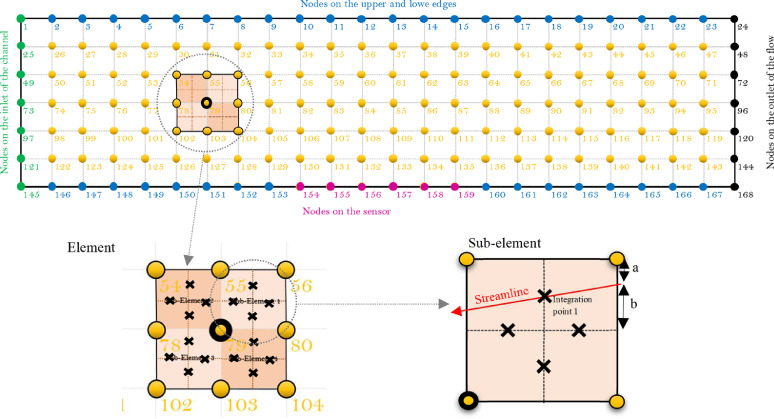
Discretization of the numerical model into nodes, elements and sub-elements, upwind point and fractions for PIS upwind scheme. The numbering represents a typical format for a channel.

Since the control-volume based finite-element method is used in the present computational code, equations are discretized in a conservative form in order to satisfy the conservations through all surfaces. For implementing the control-volume based finite-element method, first the numerical domain is discretized into nodes, elements and sub-elements, as it is shown in Fig. 3. Each element consists of four sub-elements.

For discretization, the integration of the main equations Eqs. (1)–(11) on each sub-element (control volume) is calculated. To solve the above-mentioned equations, velocity and pressure are needed at integration points (see Fig. 3). Different interpolation methods are used for generating values in integration points, since interpolation does not take the physics of the flow into account and it is not reliable for the entire range of Peclet numbers. This case is more evident in flows with high Peclet numbers. In this code, the variables in integration points are calculated with the PIS upwind method. For this method, the required variables in upstream points of flow are generated. With the values of velocity in *x* and *y* directions and coordinates of upwind points, the equation of the streamline passing by these points can be generated (Eq. (12)).

(12) $$y-y_{ip}=\frac{v}{u}\left(x-x_{ip}\right)$$

where subscript *ip* represents the integration points. Interaction of this line with edges of each element, generates the upstream and downstream points of the flow, based on the direction of the flow. Having obtained the information on the upwind points, the value of variables (φ) in the upwind position can be calculated through (Eqn 13).

(13) $$\varphi_{up_{1}=\frac{a\times \varphi_{4}+b\times \varphi_{1}}{a+b}}$$

where *a* and *b* represent the portions of an element split by the streamline, as it is shown in Fig. 3 and subscripts 1 and 4 indicate the number of sub-element. Subsequently, a 4 × 4 matrix is generated for each element (Eq. (14)), which connects the value of upwind point to 4 nodes of each element. With these values, flow can be solved by the upwind method.

(14) $$\left[\begin{array}{cccc} C_{up1,1} & C_{up1,2} & C_{up1,3} & C_{up1,4}\\ C_{up2,1} & C_{up2,2} & C_{up2,3} & C_{up2,4}\\ C_{up3,1} & C_{up3,2} & C_{up3,3} & C_{up3,4}\\ C_{up4,1} & C_{up4,2} & C_{up4,3} & C_{up4,4} \end{array}\right]\left[\begin{array}{c} \varphi_{1}\\ \varphi_{2}\\ \varphi_{3}\\ \varphi_{4} \end{array}\right]=\left[\begin{array}{c} \varphi_{up1}\\ \varphi_{up2}\\ \varphi_{up3}\\ \varphi_{up4} \end{array}\right]$$

The area of each element and sub-element (*ds_x_* and *ds_y_*) needed in the calculations of integrations over control volume are generated within the subroutine ‘Area’. At the end, for solving the main equations, the value of each element is obtained by a bilinear interpolation between the 4 sub-elements. Details of these procedures are described in the next section.

## Structure of the code

**Fig. 4:**
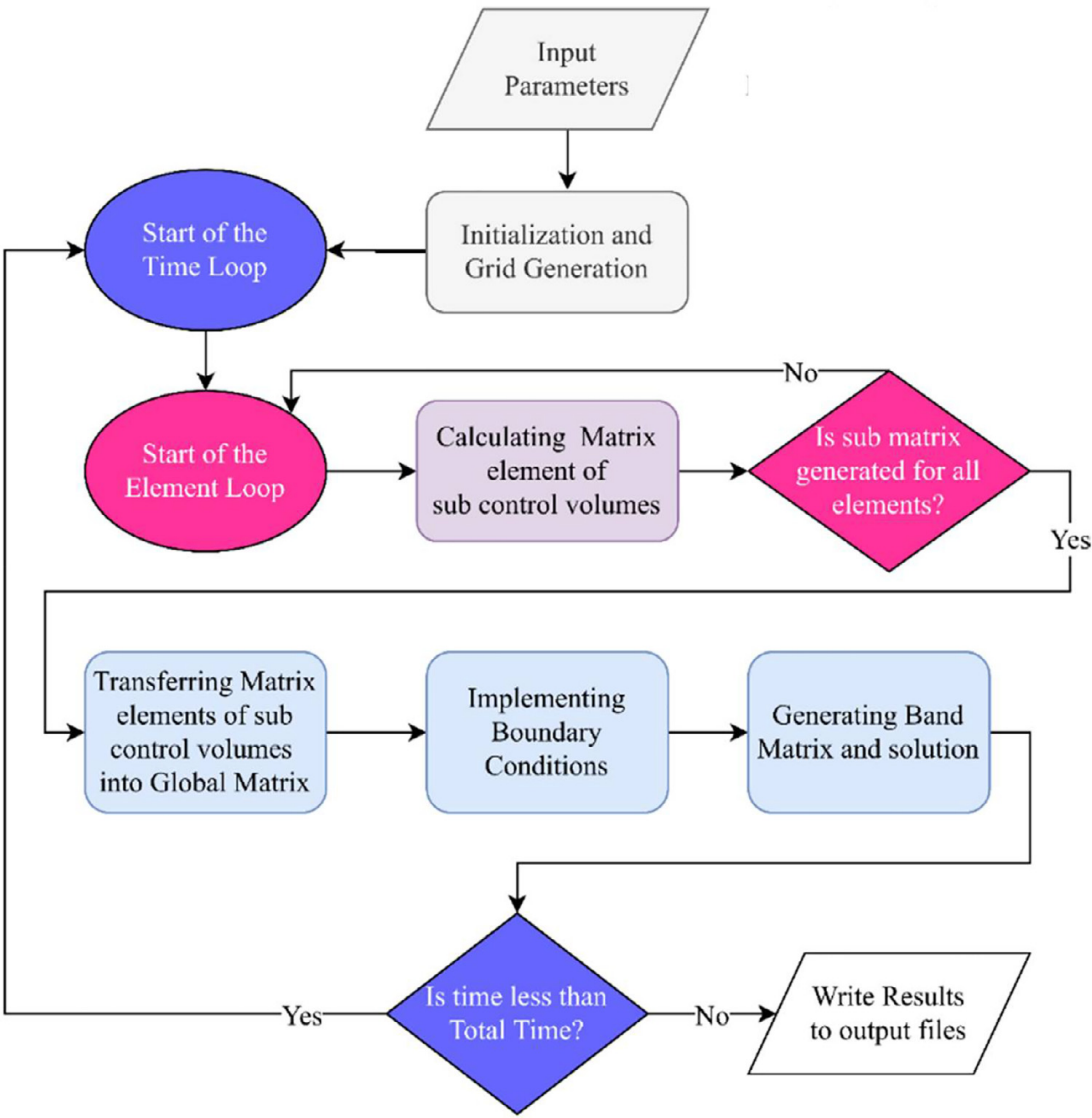
Solution diagram of the code.

The proposed numerical model can solve the microfluidic-integrated biosensor with different geometries, sensor, position of the sensor, buffer fluid and meshes. The list of these parameters is presented in Table 3. Fig. 4 and Table 4 show the structure of the proposed numerical model.

**Table 3:** The user input parameters.

Parameter	Sign	Unit		Parameter	Sign	Unit
Height of the channel	*H*	m		Flow rate	*Q*	m³s⁻¹
Length of the channel	*L*	m		Inlet concentration	*c_0_*	Mol m⁻³
Length of the sensor	*L_s_*	m		Diffusion coefficient	*D*	m^2^ s^−1^
Density of binding sites	*b_max_*	Mol m²		Number of nodes on the sensor	*N_s_*	*–*
Adsorption rate	*k_on_*	m³Mol⁻¹ s⁻¹		Number of nodes on x direction	*N_x_*	*–*
Disassociation rate	*k_off_*	s⁻¹		Number of nodes on y direction	*N_y_*	*–*

**Table 4:** The structure of the numerical model.

## Method validation

**Fig. 5:**
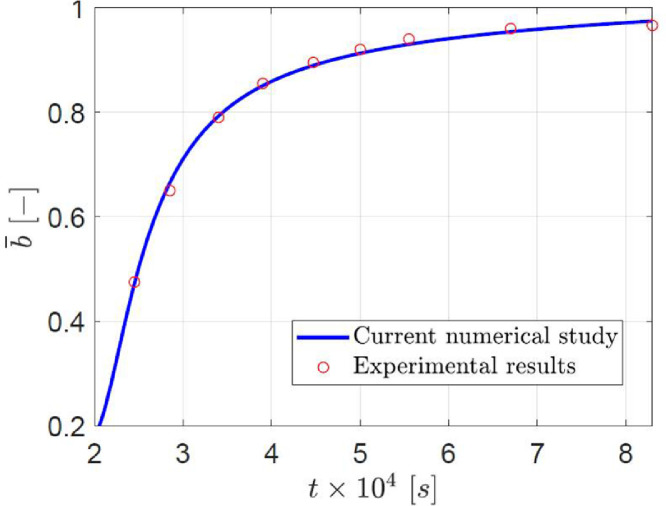
Validation of the current numerical model with the experimental data [1] for the normalised surface concentration ($\bar{b}$) over time. The surface concentration is normalised to the maximum density of binding sites on the sensor.

This model has been validated with experimental data provided by Berthier and Silberzan [1]. Experiments were done in a micro chamber with the details provide in Table 5 for different DNA strands. The buffer fluid enters the chamber while carrying the targeted molecules. This experiment takes 10^5^ s. The results are shown in Fig. 5, where good agreement is obtained, demonstrating a success in using the present methodology for modelling convective diffusive and reactive targeted molecules inside microfluidic integrated biosensors. The developed numerical model is suitable for modelling flow inside microfluidic-integrated biosensors, which is adaptable to various geometries and operating conditions. Although this version of the simulation platform has several limitations, based on the boundary conditions and assumptions defined in the code, it is capable of improvements can be made by through implementing more applicable numerical models for future studies. For instance, the concentration of the targeted molecules is assumed to be high enough for a bulk concentration, although this assumption cannot be valid for samples with low analyte concentrations. The performance of this numerical platform in other favourable applications in analysis of miniaturised biological devices such as indirect measurement of chemical detection [9], could be investigated and enhanced in future versions.

**Table 5:** Details of the validation case [1].

Parameter	Sign	Unit	Value
Flow rate	*Q*	m³s⁻¹	7 × 10⁻¹¹
Inlet concentration	*c_0_*	Mol m⁻³	2.5 × 10⁻⁶
Channel dimension	*L × H*	m × m	10^−2^ × 10⁻³
Density of binding sites	*b_max_*	Mol m²	1.668 × 10⁻⁸
Adsorption rate	*k_on_*	m³Mol⁻¹ s⁻¹	75
Disassociation rate	*k_off_*	s⁻¹	10⁻²

## Declaration of Competing Interest

The authors declare that they have no known competing financial interests or personal relationships that could have appeared to influence the work reported in this paper.
